# Supplementary material for: Life as a Categorical Information-Handling System: An Evolutionary Information-Theoretic Model of the Holobiont
Source: Biology (Basel). 2026 Jan 10;15(2):125. doi: 10.3390/biology15020125 (PMC12837587; doi:10.3390/biology15020125)
Supplement: Supplementary file 1 [file biology-15-00125-s001.zip › biology-4063961-supplementary.pdf]

Supplementary Materials Mathematical Section to "Life as a Categorical Information-Handling System: An Evolutionary Information-Theoretic Model of the Holobiont"

Antonio Carvajal-Rodríguez [1,*]

[1] Centro de Investigación Mariña (CIM), Departamento de Bioquímica, Genética e Inmunología. Universidade de Vigo, 36310 Vigo, Spain; acraaj@uvigo.es

[*] Correspondence: acraaj@uvigo.es

SECTION S1: SELECTION INFORMATION MODEL IN THE HOLOBIONT

Let p_i be the frequency of host type i and m_j the frequency of microbiome type j. Then, before selection, the expected frequency of holobionts of type $i{\times}j$ is

$$q_{ij} = p_i m_j$$

S1.1 Multiplicative selection with affinities

$$W_{ij} := W_G(i) \times W_M(j) \times a_{ij} \ \forall i, j: W_G(i) > 0, W_M(j) > 0, a_{ij} > 0,$$

$$\bar{W} := \sum p_i\, m_j W_{ij},$$

$$q'_{ij} = p_i m_j \frac{W_{ij}}{\bar{W}}.$$

S1.2 Marginal frequencies after selection

$$p'_i = \sum q'_{ij} = p_i W_G(i) \frac{A_i}{\bar{W}},$$

$$m'_j = \sum q'_{ij} = m_j W_M(j) \frac{B_j}{\bar{W}},$$

where

$$A_i := \sum m_j\, W_M(j) a_{ij},$$

$$B_j := \sum p_i\, W_G(i) a_{ij},$$

and the residual affinity with respect to the new marginal frequencies:

$$a'_{ij} := \frac{q'_{ij}}{p'_i m'_j} = a_{ij} \frac{\bar{W}}{A_i B_j},$$

because

$$q'_{ij} = p_i m_j \frac{W_{ij}}{\bar{W}} = p'_i \cdot m'_j \cdot a'_{ij} \;\Rightarrow\; p_i m_j \frac{W_{ij}}{\bar{W}} = \frac{p_i W_G(i) A_i}{\bar{W}} \cdot \frac{m_j W_M(j) B_j}{\bar{W}} \cdot a'_{ij} \;\Rightarrow\; a_{ij}/\bar{W}$$

$$= \frac{A_i B_j}{\bar{W}^2} a'_{ij},$$

so

$$a'_{ij} = \frac{a_{ij} \cdot \bar{W}}{A_i B_j}.$$

S1.3 General information and partitioning

Taking $Z = log(W/\bar{W})$ the average change of Z due to selection is

$$J := \sum \left(q'_{ij} - q_{ij}\right) log \frac{q'_{ij}}{q_{ij}},$$

and we obtain the partition

$$J = J_G + J_M + I_{assoc},$$

where

$$J_G = \sum (p'_i - p_i)\, log \frac{p'_i}{p_i} = \sum (p'_i - p_i)\, log \frac{W_G(i) A_i}{\bar{W}},$$

$$J_M = \sum \left(m'_j - m_j\right) log \frac{m'_j}{m_j} = \sum \left(m'_j - m_j\right) log \frac{W_M(j) B_j}{\bar{W}},$$

$$I_{assoc} := \sum \left(q'_{ij} - q_{ij}\right) log\left(a'_{ij}\right).$$

S1.3.2 Verification

$$q_{ij} = p_i m_j,\; q'_{ij} = p'_i m'_j a'_{ij},$$

$$log \frac{q'_{ij}}{q_{ij}} = log \frac{p'_i}{p_i} + log \frac{m'_j}{m_j} + log\left(a'_{ij}\right).$$

Then we can express J as

$$J = \sum(q'_{ij} - q_{ij}) \cdot \left[log\frac{p'_i}{p_i} + log\frac{m'_j}{m_j} + log(a'_{ij})\right].$$

We separate it into three sums and note that

$$\sum(q'_{ij} - q_{ij}) = p'_i - p_i$$

and

$$\sum(q'_{ij} - q_{ij}) = m'_j - m_j,$$

we obtain

$$J_G := \sum(p'_i - p_i)\, log\frac{p'_i}{p_i},$$

$$J_M := \sum(m'_j - m_j)\, log\frac{m'_j}{m_j},$$

$$I_{assoc} := \sum(q'_{ij} - q_{ij})\, log(a'_{ij}) = \sum(q'_{ij} - q_{ij})\, log\frac{a_{ij}\cdot\bar{W}}{A_i\cdot B_j}.$$

Multiplicative selection moves the marginals (J_G, J_M) through W_G and W_M, while the host-microbiota pairing structure is captured in I_{assoc} via affinity a_{ij} versus their row/column averages (A_i, B_j).

SECTION S2: CATEGORICAL MODEL OF INFORMATION HANDLERS WITH (*M*,*R*)-CLOSURE AND INFORMATIONAL FOLDS

S2.1 Quiver and Lineage Categories R_α (IH)

S2.1.1 Basic Quiver (Directed Graph)

An initial object $X(0)$ is defined. From $X(0)$, variants $A(1)$, $A(2)$,…, $A(t)$; $B(1)$, $B(2)$,…, $B(t)$;… are generated through replication with or without mutation, where t represents discrete generations from the initial configuration. For each linage $\alpha \in \{A, B, C, \ldots\}$: at each step t the classes $\alpha_0(t), \alpha_1(t), \ldots, \alpha_t(t)$, coexist, representing the accumulated substates of the lineage. This step-by-step dynamic can be viewed as a discrete coalgebra, that is, a rule that, given the current state, produces the next one. Biologically, a coalgebra describes how a living system transforms or replicates, generating its future state from the present.

S2.1.2 Definition (Lineage Category R_α)

Level-0 Objects (Informational Matter)

$$Ob_0(R_\alpha) = \{X(0), \alpha(t), \dots\}.$$

These are the concrete information handlers. *X*(0) is the original handler from which all others derive. Each α(*t*) represents a discrete material state of the lineage (molecule, replicator, population, or configuration). The morphisms between these objects describe material transformations: replication, mutation, or reproduction.

Level-1 Objects (Functional)

$$Ob_1(R_\alpha) = \{[X(0), \alpha(1)]\} \cup \{[\alpha(t), \alpha(t+1)] | t \geq 1\}.$$

For each pair (α(t), α(t+1)), there exists a second level object [α(*t*), α(*t*+1)] representing the functional space of the possible transformations from α(*t*) in α(*t*+1):

$$[\alpha(t), \alpha(t+1)] \in Ob(R_\alpha).$$

Formally, [α(*t*), α(*t*+1)] is an internal object of the category, but biologically it represents the rules, enzymes, or functions that carry out the transformation from one material state to another.

S2.1.3 Morphisms (arrows)

Each elementary morphism of lineage α is denoted

$$f_{\alpha,t}: \alpha(t) \to \alpha(t+1),$$

where α(*t*) is the information handler at step *t* (informational matter), and $f_{\alpha,t}$ Is the combined action of replication and mutation producing the new IH α(*t*+1). Here, it is not necessary to specify internal subtypes *s*, since the arrow refers to the entire set or distribution of the lineage at time *t*.

Additionally, two internal arrows are defined to link the material level with the functional one:

$$\rho_t: \alpha(t) \to [\alpha(t), \alpha(t+1)], ev_t: [\alpha(t), \alpha(t+1)] \to \alpha(t+1).$$

The arrow ρ_t assigns to each resulting state α(*t*+1) the mechanism that can reproduce it in the future; it is an abstraction of the production of enzymes or functions enabling replication. Its composition with ev_t generates the effective transformation of the system:

$$f_{\alpha t} = ev_t \circ \rho_t.$$

Altogether, the system produces the means that ensure its own future replication, thus fulfilling the condition of discrete (M,R) closure:

$$(M,R)\colon \alpha(t) \overset{\rho_t}{\rightarrow} [\alpha(t),\alpha(t+1)] \overset{ev_t}{\rightarrow} \alpha(t+1).$$

See the Reproduction section S2.6.2 for more details on the replication.

S2.2 Supercategory *R* (Category of Categories)

The set of all lineages forms a supercategory *R*, whose objects are the categories R_α and whose morphisms are functors between them. These morphisms represent processes of reproduction or interaction between lineages (e.g., hybridization, gene transfer, symbiosis, etc.). The natural transformations between these functors, representing coherent changes, for instance, in modes or preferences of pairing, endow *R* with the structure of a bicategory, in which the composition of processes is preserved not strictly but up to natural equivalence, that is, up to a structural correspondence that maintains functional coherence among different modes of interaction.

In other words, the composition of processes is preserved in such a way that the biological and evolutionary coherence of the system is maintained.

$$Ob(R) = \{R_\alpha \mid \alpha \in \Lambda\},$$

$$Mor(R) = \{F\colon R_\alpha \rightarrow R_\beta \mid F \text{ is a functor}\}.$$

S2.3 Populations: Deterministic Functor to Set

Each object at state *s* in lineage α is connected to the set of copies it contains. To do this, for each lineage α, we define a deterministic population functor:

$$P_\alpha\colon R_\alpha \rightarrow \text{Set},$$

which assigns to each object $\alpha_s(t)$ a set of instances of the information handler, and to each morphism a deterministic function between sets.

S2.3.1 Objects

Each abstract object $\alpha_s(t) \in Ob(R_\alpha)$is realized as a set of temporal instances given by P_α $(\alpha(s); t)$.

S2.3.2 Morphisms

For each elementary morphism $f_{\alpha,s}$ in R_α,

$$f_{\alpha,s}: \alpha(s) \rightarrow \alpha(s+1),$$

the functor

$$P_\alpha(f_{\alpha,s}; t): P_\alpha(\alpha(s); t) \rightarrow P_\alpha(\alpha(s+1); t+1),$$

associates the population of subtype $\alpha_s(t)$ at time *t* with the population of subtype $\alpha_{s+1}(t+1)$ at time *t*+1. The increment from *s* to *s*+1 reflects the appearance of at least one new variant during replication of the subtype, so that the time step also incorporates the genetic innovation accumulated within the lineage.

S2.3.3 Functorial Property

$$P_\alpha(id_{\alpha(s)}) = id_{P_\alpha(\alpha(s);t)},$$

$$P_\alpha(f_{\alpha,s+1} \circ f_{\alpha,s}) = P_\alpha(f_{\alpha,s+1}) \circ P_\alpha(f_{\alpha,s}).$$

S2.3.4 Cardinalities and Frequencies per Lineage

Let

$$N_{\alpha,s}(t) = |P_\alpha(\alpha(s); t)|$$

denote the number of IHs of subtype *s* that are still present at time *t*; where

$$P_\alpha(f_{\alpha,s}; t)$$

denote the replication/mutation function.

Then define

$$N_\alpha^{tot}(t) = \sum N_{\alpha,s}(t),$$

$$p_{\alpha,s}(t) = \frac{N_{\alpha,s}(t)}{N_\alpha^{tot}(t)},$$

$$\sum p_{\alpha,s}(t) = 1,$$

where

$p_{\alpha,s}(t)$ denote the current frequency of that subtype.

S2.3.5 Functional Summary

The functor P_α links the categorical structure of the lineage

$$R_\alpha = \{\alpha(0), \alpha(1), \dots, \alpha(T)\},$$

with its concrete realization in population sets

$$P_\alpha(\alpha(s); t) \subseteq \text{Set},$$

allowing to define

$$N_{\alpha,s}(t+1) = r_{\alpha,s}(t) N_{\alpha,s}(t),$$

where $r_{\alpha,s}(t)$ is the effective growth or persistence rate of each type s, including the net effect of entries and exits due to mutation or extinction of that type.

S2.4 Intra-Lineage Replicator Dynamics

Given the set of substates {α(0),α(1),…,α(t)} of lineage α at step t and letting $p_{\alpha s}$ denote the current frequency of subtype s at that time, the frequency in the next generation, considering only differences in the replication rate (i.e., with no mutation or other transmission effects), is given by

$$p_{\alpha,s}(t+1) = \frac{p_{\alpha,s}(t) \cdot r_{\alpha,s}(t)}{\bar{r}_\alpha(t)}, \qquad \text{(S2.1)}$$

where

$$\bar{r}_\alpha(t) = \sum r_{\alpha,s}(t) \cdot p_{\alpha,s}(t),$$

is the mean replication rate in the population.

S2.4.1 Consistency between Counts and the Replicator Equation

Assuming that, in the transition from t to t+1, new objects $\alpha_{t+1}(t+1)$ are generated, belonging to a new class $s = t+1$, and that some classes $s \le t$ may disappear (i.e., become the empty set), then

$$N_{\alpha,s}(t+1) = \sum N_{\alpha,i}(t) \cdot r_{\alpha,i}(t) \cdot Q_{i \to s}(t), \qquad \text{(S2.2)}$$

where Q is a stochastic transition matrix that captures the probabilities of transition between states.

Thus we obtain

$$N_\alpha^{tot}(t+1) = \sum N_{\alpha,s}\,(t+1) = \sum\sum N_{\alpha,i}\,(t)\cdot r_{\alpha,i}(t)\cdot Q_{i\to s}(t)$$

$$= \sum N_{\alpha,i}\,(t)\cdot r_{\alpha,i}(t)\cdot \sum Q_{i\to s}\,(t),$$

so

$N_\alpha^{tot}(t+1) = \sum N_{\alpha,i}\,(t)\cdot r_{\alpha,i}(t) = N_\alpha^{tot}(t)\cdot \bar{r}_\alpha(t)$because $\sum Q_{i\to s}\,(t) = 1\forall i$,

and dividing both sides of (S2.2) by $N_\alpha^{tot}(t+1)$and substituting $N_\alpha^{tot}(t+1) = \bar{r}_\alpha(t)\cdot N_\alpha^{tot}(t)$ into the right-hand side, we obtain

$$p_{\alpha,s}(t+1) = \frac{\sum p_{\alpha,i}(t)\cdot r_{\alpha,i}(t)\cdot Q_{i\to s}(t)}{\bar{r}_\alpha(t)}.$$

To recover the replicator equation (S2.1), we define an effective replication rate that incorporates the effect of mutation:

$$r_{\alpha,s}^{(eff)}(t) = \frac{N_{\alpha,s}(t+1)}{N_{\alpha,s}(t)} = r_{\alpha,s}(t)Q_{s\to s}(t) + \sum{}_{i\neq s}\frac{N_{\alpha,i}(t)}{N_{\alpha,s}(t)}r_{\alpha,i}(t)Q_{i\to s}(t),$$

passing to frequencies,

$$r_{\alpha,s}^{eff}(t) = r_{\alpha,s}(t)Q_{s\to s}(t) + \sum r_{\alpha,i}\,(t)\left[Q_{i\to s}\,(t)/p_{\alpha,s}\,(t)\right]p_{\alpha,i}(t).$$

The term $r_{\alpha,s}^{eff}(t)$ meassures the effective fitness of class s.

Thus the replicator equation (S2.1) now becomes

$$p_{\alpha,s}(t+1) = \frac{p_{\alpha,s}(t)\cdot r_{\alpha,s}^{eff}(t)}{\bar{r}_\alpha^{eff}(t)}, \qquad \text{(S2.3)}$$

where

$$\bar{r}_\alpha^{eff}(t) = \sum p_{\alpha,u}\,(t)\cdot r_{\alpha,u}(t).$$

In this way, the effective replication rate measures the total change factor of the population, including both the descendants that remain in the same class and those that arrive from other classes.

S2.5 Functional or Information Functor with Two Channels J_r and J_{rt}

The information functor Info_α acts on the replication morphisms $f_{\alpha,t}$, but its value is computed as the Jeffreys divergence between the frequency distributions induced by these morphisms through the population functo P_α. The functor can be decomposed into two channels: the selective channel

$Info_\alpha^{(r)}$, which measures the information due exclusively to selection, and the total channel $Info_\alpha^{(rt)}$, which also includes the effects of transmission.

S2.5.1 Basic notation

$$p(t) = p_\alpha(t) = \left(p_{\alpha,0}(t), p_{\alpha,1}(t), \dots\right),$$
$$\bar{r}_\alpha(t) = \sum p_{\alpha,s}(t) \cdot r_{\alpha,s}(t),$$
$$r'_{\alpha,s}(t) = r_{\alpha,s}(t)/\bar{r}_\alpha(t),$$
$$r'(t) = \left(r'_{\alpha,0}(t), r'_{\alpha,1}(t), \dots\right).$$

S2.5.2 Update by Selection

For each state *s* at time *t* ($s \le t$), the state at *t*+1 is updated according to (S2.1), here written as

$$(p_r)_s(t+1) = r'_{\alpha,s}(t) p_{\alpha,s}(t).$$

S2.5.3 Update by Selection + Transmision

Here, transmission includes the effects of mutation, migration, or class flow, absorbed into an effective replication rate. For each state *s* at time *t* ($s \le t$), the state at *t*+1 is updated according to (S2.3).

S2.5.4 Informational Functionals

(a) Purely Selective Information

$$Info_\alpha^{(r)}(f_{\alpha,t}) = J\left(p_r(t+1), p(t)\right) = \sum \left((p_r)_s(t+1) - p_{\alpha,s}(t)\right) ln \frac{(p_r)_s(t+1)}{p_{\alpha,s}(t)}.$$

(b) Total Information (Selection + Transmission)

$$Info_\alpha^{(rt)}(f_{\alpha,t}) = J\left(p(t+1), p(t)\right) = \sum \left(p_{\alpha,s}(t+1) - p_{\alpha,s}(t)\right) ln \frac{p_{\alpha,s}(t+1)}{p_{\alpha,s}(t)},$$

where $J \ge 0$ and $J = 0$ iff the distributions at *t* and *t*+1 are identical.

(c) Transmissional (Non-Selective) Component

For each step *t*, we obtain the partition:

$$Info_\alpha^{(rt)} = Info_\alpha^{(r)} + T_{trans}\left(\overrightarrow{(p)}, \overrightarrow{(r)}, Q\right),$$

where

$$T_{trans}(f, r, Q) = \sum f_{\alpha,s}(t) \left[\left(\frac{r_{\alpha,s}^{eff}(t)}{\bar{r}_\alpha(t)} - 1\right) ln \frac{r_{\alpha,s}^{eff}(t)}{\bar{r}_\alpha(t)} - \left(\frac{r_{\alpha,s}(t)}{\bar{r}_\alpha(t)} - 1\right) ln \frac{r_{\alpha,s}(t)}{\bar{r}_\alpha(t)}\right]$$

is the component due to transition effects, as a function of frequencies, replication rates, and the transition matrix *Q*.

If $Q = I$ (identity) then $T_{trans} = 0$ and $J_{rt} = J_r$ the causes of change are then purely selective.

S2.5.5 Handling of Zeros

In the case of appearance of a new class $p_{\alpha,s}(t)=0$, $p_{\alpha,s}(t+1)>0$ (e.g. $s = t+1$), the corresponding term in J_{rt}

$$\big(p_{\alpha,s}(t+1) - 0\big) ln p_{\alpha,s}\,(t+1)/0$$

diverges. Conceptually, this can be interpreted as *J* jumping to infinity when innovation (new information) arises.

In the case of disappearance of a class, $p_{\alpha,s}(t)>0$, $p_{\alpha,s}(t+1)=0$, the object transitions to the empty set, conceptually not problematic, as it represents the loss of an informational channel.

In both cases, the problem arises only when computing *J* explicitly. To handle this, we apply a regularization, assuming a smoothed version of the distributions such that the support is complete (no zeros). For both distributions, we assume

$$p^{\varepsilon} = (1-\varepsilon)p + \varepsilon/K \quad (1 >> \varepsilon > 0),$$

where *K* is the number of classes.

Thus, for any *J*, we can write

$$J_{\varepsilon}\big(p(t+1), p(t)\big) = \textstyle\sum_s \big(p_s^{\varepsilon}(t+1) - p_s^{\varepsilon}(t)\big)\, ln \frac{p_s^{\varepsilon}(t+1)}{p_s^{\varepsilon}(t)}.$$

S2.5.6 Local Fold in R_α

$$Info_\alpha^{(rt)}\big(f_{\alpha,t}\big) = J\big(p_\alpha(t+1), p_\alpha(t)\big),$$

$$Info_\alpha^{(rt)}(T) = \textstyle\sum Info_\alpha^{(rt)}\,\big(f_{\alpha,t}\big),$$

measures the total information generated by selection and transmission within lineage α.

S2.6 Reproduction *M*, Mating Fitness *m* and J_{repr}

S2.6.1 Reproductive Operator (Categorical Product)

Reproduction is modeled as a binary operation (a reproductive bifunctor) between lineage objects

$$M: \alpha_s(t) \times \beta_{s'}(t) \rightarrow (\alpha\beta)(t+1),$$

where M maps pairs of parental lineages to the lineage (or set of offspring lineages) they produce.

S2.6.2 Pure Replication

In the case of pure replication, reproduction occurs within a single lineage and involves no pairing or combination between different entities. Two complementary types of morphisms can be distinguished:

(a) Identity morphism (structural invariance)

$$id_{\alpha(t)}: \alpha(t) \rightarrow \alpha(t).$$

This morphism is structural, not temporal. It represents the self-preservation or instantaneous maintenance of the informational handler in its current state, without invoking any passage of time. Biologically, it expresses the potential of the system to remain stable, preserving its organization and information content, within a given temporal frame.

(b) Replication morphism (causal process)

$$f_{\alpha,t}: \alpha_s(t) \rightarrow \alpha_{s'}(t+1).$$

This morphism represents the discrete temporal transition from step *t* to *t*+1. It may or may not involve a change in the internal state *s* depending whether replication occurs without mutation (*s*'=*s*), preserving the internal configuration or wheher replication occurs with mutation or variation (*s*'≠*s*), producing a new subtype within the lineage. The morphism $f_{\alpha,t}$ therefore captures the causal process by which the system generates its next configuration, either identical or modified, maintaining lineage continuity while potentially introducing informational change.

S2.6.3 Intra-Lineage Reproduction

Within a lineage, reproduction

$$M_{\alpha;s,s'}: \alpha_s(t) \times \alpha_{s'}(t) \rightarrow \alpha_{(s \boxplus s')}(t+1)$$

is represented by the discrete combination rule $\boxplus$, that is, a function

$$\gamma: S \times S \rightarrow S,$$

that determines the type of offspring:

$$\alpha_s(t) \times \alpha_{s+1}(t) \rightarrow \alpha_{\gamma(s,s')}(t+1).$$

For example,

$$\alpha_s(t) \times \alpha_{s+1}(t) \rightarrow \alpha_{s+1}(t+1),$$

which represents that the lineage state *s*+1 dominates, or

$$\alpha_s(t) \times \alpha_{s'}(t) \rightarrow \alpha_{s''}(t+1)$$

represents the generation of a new subtype within lineage α.

S2.6.4 Inter-Lineage Reproduction

Between distinct lineages α and β, reproduction follows a cross-lineage generation rule

$$M_{\alpha,\beta}: \alpha_s(t) \times \beta_{s'}(t) \rightarrow (\alpha\beta)_{\gamma(s,s')}(t+1),$$

where γ(s, s') generates combined states between lineages.

S2.6.5 Mating frequencies

Let $p_i(t)$ and $p_j(t)$ denote the frequencies of object *i* in lineage α and object *j* in lineage β at time *t*, respectively. The probability of random mating between them is:

$$q_{ij}(t) = p_i(t)p_j(t)$$

and the probability of mating influenced by mating fitness m_{ij} is:

$$q'_{ij}(t) = q_{ij}(t)m_{ij}(t)/M(t),$$

where

$$M(t) = \sum q_{ij}(t)m_{ij}(t)$$

is the mean mating fitness.

After mating, the replication rates depend on the corresponding reproductive rule:

$$\phi_{\alpha,\beta}: r_\alpha \times r_\beta \rightarrow r_{\alpha\beta}^{post} \circ \hat{m}_{\alpha,\beta}.$$

In general form:

$$r_{ij}^{post}(t) = G\left(r_{\alpha,i}(t), r_{\beta,j}(t)\right)$$

and, in specific cases:

$$G_L(x,y) = x, G_R(x,y) = y, G_{mean}(x,y) = (x+y)/2,$$

$$G_{geom}(x,y) = \sqrt{xy}, G_\lambda(x,y) = \lambda x + (1-\lambda),$$

$$G_{hibrid}(x,y) = I_{xy}\,(x+y)/2,$$

where $I_{xy} \in [0,+\infty)$ is a factor representing sexual isolation, where $I_{xy} = 0$ corresponds to complete isolation.

S2.6.6 Reproductive Fold (Jeffreys Divergence in Pair Space)

The deviation from random mating is measured by the Jeffreys divergence in the space of pairs:

$$J_{repr}\big(q'(t), q(t)\big) = \sum \left(q'_{ij}(t) - q_{ij}(t)\right) ln \frac{q'_{ij}(t)}{q_{ij}(t)}.$$

This measure can, in turn, be separated into additive components of male and female sexual selection and an assortative mating component [1].

S2.6.7 Decomposition by Lineage Relationship

In this formulation, reproduction is decomposed into three informational channels, corresponding to the total reproductive channel J_{repr}, the intra-lineage channel $J_{repr,intra}$, and the inter-lineage channel $J_{repr,inter}$.

Intra-lineage (within α):

$$J_{repr}^{intra,\alpha} = \sum\big(q'_{ij} - q_{ij}\big)\, ln \frac{q'_{ij}}{q_{ij}}.$$

Inter-lineage (between α and β):

$$J_{repr}^{inter,\alpha,\beta} = \sum\big(q'_{ij} - q_{ij}\big)\, ln \frac{q'_{ij}}{q_{ij}},$$

where the indices *i*, *j* range over the coexisting substates at step *t*.

In this partition of reproductive information, we distinguish the total, intra-lineage, and inter-lineage channels, each defined over its own normalized distribution. Since the normalizations differ, the corresponding divergences are not additive, but each preserves probability consistency and an informational interpretation within its respective domain.

S2.7 Higher Level: Global Fold in *R*

The global fold integrates the total information generated by replication, transmission, and reproduction.

S2.7.1 Total information at t

$$Info_{Total}(t) = J_{repr}(q'(t), q(t)) + \sum J_{rt}\left(f_\alpha(t+1), f_\alpha(t)\right).$$

S2.7.2 Global Information over T: Purely Selective Option

$$Info^{(r)}_{Global}(T) = \sum J_r\left(f_\alpha(t+1), f_\alpha(t)\right).$$

This expression measures the accumulated information due solely to selection across all lineages and discrete time steps.

S2.7.3 Global Information over T: Total Option (Selection + Transmission)

$$Info^{(rt)}_{Global}(T) = \sum J_{rt}\left(f_\alpha(t+1), f_\alpha(t)\right).$$

This includes selection plus the transmission effects (mutation, recombination, migration) accumulated through time and across all lineages.

S2.7.4 Explicit Decomposition of the Transmission Channel

$$Info^{rt}_{Global} = Info^{r}_{Global} + \sum T_{Trans,\alpha}\,(t),$$

$$\Delta Info^{Global}_{trans}(T) = Info^{(rt)}_{Global}(T) - Info^{(r)}_{Global}(T) = \sum \Delta\, Info_{Trans}(\alpha, T)$$

represents the total information associated with non-selective transmission effects.

S2.7.5 Total Global Information in T

$$Info^{Total}_{Global}(T) = \sum Info_{Total}\,(t),$$

which captures the complete informational content accumulated by the system due to selection, transmission, and reproduction over the interval $[0,T)$.

SECTION S3: MULTICOMPONENT MICROBIOME DECOMPOSITION AND THE GENERALIZED INFORMATION PARTITION

In many biological scenarios, the microbiome associated with a host is not adequately represented as a single lineage, but rather as a structured assemblage of multiple microbial components (e.g., phyla, functional guilds, or ASV-defined sublineages). To capture this structure, we extend the holobiont model by decomposing the microbiome into K components, each with its own internal dynamics. A holobiont state is therefore indexed by a host type i and a vector of microbiome types $(j_1,\ldots,j_K)$, where j_k denotes the state of the k-th microbiome component.

Let the joint pre-selection frequency be

$q_{i,j_1,\dots,j_K}(t)$.

S3.1 Fitness decomposition

We assume a multiplicative decomposition of absolute holobiont fitness

$$W_{i,j_1,\dots,j_K}(t) = W_H(i,t) \cdot \prod W_{M_k}(j_k,t) \cdot \prod a_{ij_k}^{(k)}(t),$$

where

$W_H(i,t)$ is the host contribution,

$W_{M_k}(j_k,t)$ is the contribution of microbiome component *k* and

$a_{ij_k}^{(k)}(t)$ is the host–component-*k* association factor.

Under selection, the next-generation frequences follow the standard update:

$$q'_{i,j_1,\dots,j_K}(t) = q_{i,j_1,\dots,j_K}(t) \cdot \frac{W_{i,j_1,\dots,j_K}(t)}{\bar{W}(t)}.$$

S3.2 Relative log-fitness and informational partition

Define the relative log-fitness

$$Z_{i,j_1,\dots,j_K}(t) := log \frac{W_{i,j_1,\dots,j_K}(t)}{\bar{W}(t)}.$$

Because relative log-fitness differs from absolute log-fitness by a constant

$$-log(\bar{W})$$

and since

$$\sum_{i,j_1,\dots,j_K}(q' - q) = 0,$$

the informational change due to selection is

$$J = \sum \left(q'_{i,j_1,\dots,j_K} - q_{i,j_1,\dots,j_K}\right) Z_{i,j_1,\dots,j_K}.$$

Using the multiplicative structure of the fitness, Z decomposes additively as:

$$Z_{i,j_1,\dots,j_K} = Z_H(i) + \sum Z_{M_k}(j_k) + \sum Z_{assoc,k}(i,j_k),$$

where

$$Z_H(i) := logW_H(i),$$

$$Z_{M_k}(j_k) := logW_{M_k}(j_k),$$

$$Z_{assoc,k}(i, j_k) := loga_{ij_k}^{(k)}.$$

By linearity this yields the multicomponent informational partition

$$J = J_H + \sum J_{M_k} + I_{assoc},$$

where

$$J_H = \sum_i (p'_i - p_i)\, Z_H(i),$$

$$J_{M_k} = \sum (m'_{k,j_K} - m_{k,j_K})\, Z_{M_k}(j_k),$$

and

$$I_{assoc} = \sum (q'_{i,j_1,\dots,j_K} - q_{i,j_1,\dots,j_K}) \sum Z_{assoc,k}\,(i, j_k).$$

These expressions are obtained from the joint form by summing over the microbiome (for J_H) or over all other indices (for J_{Mk}), using the fact that host marginals are given by

$$p_i = \sum q_{i,j_1,\dots,j_K}\,;\; p'_i = \sum q'_{i,j_1,\dots,j_K}$$

and microbiome marginals

$$m_{k,j_k} = \sum q_{i,j_1,\dots,j_k,\dots,j_K}\,;\; m'_{k,j_k} = \sum q'_{i,j_1,\dots,j_k,\dots,j_K},$$

where the summation runs over all indices except j_k.

Thus, the total information divides into a host component, a sum of microbiome components, and a multicomponent association term.

S3.3 Partition of the multicomponent association term

To further decompose the association term into a pure association and an interaction component, we follow the approach used in Carvajal-Rodríguez [2] and in Section 7.4.

Let define the independence reference:

$$r_{i,j_1,\dots,j_K} := p'_i \cdot \prod m'_{k,j_k},$$

that is, the independent distribution matching the post-selection marginals of q'.

As usual:

$$q' - q = (q' - r) + (r - q)$$

and

$$log\frac{q'}{q} = log\frac{q'}{r} + log\frac{r}{q}.$$

Expanding the Jeffreys divergence

$$J = \sum(q' - q)\, log\frac{q'}{q}$$

with these identities gives:

$$J = T_1 + T_2 + T_3 + T_4,$$

where

$$T_1 = \sum(r - q)\, log\frac{r}{q},$$

$$T_2 = \sum(r - q)\, log\frac{q'}{r},$$

$$T_3 = \sum(q' - r)\, log\frac{r}{q},$$

$$T_4 = \sum(q' - r)\, log\frac{q'}{r}.$$

As in the two-component case, the term $T_3 = 0$ because q' and r share the same marginal distributions.

Therefore,

$$J = \left(J_H + \sum J_{M_k}\right) + J_{assoc} + E_{multi}, \qquad \text{(S3.1)}$$

where

$$T_1 = J_H + \sum J_{M_k},$$

$$J_{assoc} := T_4 = \sum(q' - r)\, log\frac{q'}{r},$$

and

$$E_{multi} := T_2 = \sum(r - q)\, log\frac{q'}{r}.$$

Finally:

$$I_{assoc} = J_{assoc} + E_{multi}.$$

Here, J_{assoc} generalizes the "choice" component of the two-dimensional holobiont case and plays the role analogous to the J_{PSI} term in mating models, in the sense that it quantifies the information contained in the full host-multicomponent microbiome association pattern, once the marginal changes have been accounted for. The term E_{multi} is the interaction residual; it captures the non-additive information arising from the simultaneous presence of marginal and joint-structure changes, and constitutes the natural extension of both the E term [1,2] and the holobiont interaction component E_{holo}.

Equation (S3.1) reduces to the two-component holobiont model when $K = 1$, and to the mating-based partitions when the two interacting components correspond to the sexes rather than to host and microbiome.

REFERENCES CITED IN THIS SUPPLEMENTARY MATERIAL

1. Carvajal-Rodríguez, A. Non-Random Mating and Information Theory. *Theoretical Population Biology* **2018**, *120*, 103–113, doi:10.1016/j.tpb.2018.01.003.
2. Carvajal-Rodríguez, A. Unifying Quantification Methods for Sexual Selection and Assortative Mating Using Information Theory. *Theoretical Population Biology* **2024**, *158*, 206–215, doi:10.1016/j.tpb.2024.06.007.
